# Risk factors, types, and neuroimaging findings in Children with Cerebral Palsy

**DOI:** 10.12669/pjms.38.7.6175

**Published:** 2022

**Authors:** Sabeen Abid Khan, Sidra Talat, Munir Iqbal Malik

**Affiliations:** 1 Dr. Sabeen Abid Khan, Assistant Professor, Pediatrics, Shifa College of Medicine, Shifa Tameer-e-Millat University, H-8 Islamabad, Pakistan; 2 Dr. Sidra Talat, Senior Registrar, Pediatrics, Shifa College of Medicine, Shifa Tameer-e-Millat University, H-8 Islamabad, Pakistan; 3 Prof. Dr. Munir Iqbal Malik, Professor of Pediatrics, Shifa College of Medicine, Shifa Tameer-e-Millat University, H-8 Islamabad, Pakistan

**Keywords:** Cerebral Palsy, Children, Neuroimaging, Risk Factors, Types

## Abstract

**Objectives:**

Cerebral palsy is a major cause of neurodisability in children in Pakistan. The study aims to evaluate the risk factors, types and neuroimaging findings in children with cerebral palsy.

**Methods:**

All children diagnosed with cerebral palsy, between 1-16 years presenting to Shifa community health center were enrolled from January 2020 to July 2021. Informed consent was taken from parents.

**Results:**

A total of 89 patients were included, 62 (69.7%) male and 27 (30.3%) females. Mean age was 4.4 ± 2.8 years. Majority of babies were born at term 74 (84%) and 15 were preterm (16%). Most of the patients were born in hospital 55 (62%), 13 (14%) were born at home. Mean birth weight was 2.3 ± 0.3. Consanguinity was present in 56 (62.9%). Birth asphyxia 38 (42.7%) was the most common cause of cerebral palsy. Maternal antenatal risk factors identified were anemia 13 (14.6%), PIH (9%) infections (6%) were significant risk factors (<0.05). Neuroimaging was done in 37 (38.7%) of the patients only.

**Conclusion:**

Male gender is more affected in our cohort. Maternal anemia, pregnancy induced hypertension and low birth weight are significant modifiable risk factors. Prevention of these can reduce the incidence of cerebral palsy.

## INTRODUCTION

Cerebral palsy is a major cause of lifelong neurodisability worldwide.^1^ It is defined as a group of permanent disorder of posture and movement resulting from a non-progressive disturbance which occurred in fetal or infant brain.^2^ It is the most common cause of motor disability in pediatric age group, with incidence of 1-6/1000 live births. Prevalence of cerebral palsy is unknown in Pakistan due to lack of a national registry. Only a few studies are available from Pakistan among which study done in Swabi district of KPK showed a prevalence of 2.2/1000.^3^ Another study done in the sub-districts of Sukkur in Sind showed prevalence of cerebral palsy of 1.12 out of 1000 children younger than five years.^4^ However, in a country with a high under five mortality rate this might not be truly reflective of the prevalence of CP.^5^ Universally the prevalence of cerebral palsy remains static despite improvement in antenatal and newborn care. The diagnosis of cerebral palsy is as late as 5 years in low resource countries as compared to 1-2 years in high income countries.^6^

The etiology of cerebral palsy is multifactorial.^7^ Identifying the specific etiology is important for management, prognosis, and genetic counselling and recurrence risk. Antenatal and perinatal risk factors studied worldwide include birth asphyxia, prematurity, birth trauma, low birth weight, kernicterus, intracranial bleed, CNS infections, and sepsis and brain malformations.^8^ Maternal risk factors include maternal obesity, smoking, alcohol consumption and infection during pregnancy.^9^ There are differences observed between different parts of the world with prematurity more common from developed countries and birth asphyxia from developing world.^10^

Topographical classification of cerebral palsy is used internationally to identify the motor deficit.^11^ Neurological types of cerebral palsy were spastic, ataxic, dyskinetic and hypotonic. Spastic variety was further classified topographically into quadriplegic, diplegic, hemiplegic and monoplegic.

The motor disorders may be associated with disturbance of sensation, perception, cognition, behavior, seizures and secondary musculoskeletal problems.^12^ Although diagnosis of cerebral palsy is mainly clinical, the American Academy of Neurology recommends all cases of cerebral palsy should undergo neuroimaging at least once, preferably MRI because of better detection rates.^13^ Neuroimaging plays a key role in clarifying etiology, determining the nature and timing of brain lesion, establishing prognosis, for the assessment of recurrence risk and genetic counselling of families and to limit unnecessary investigations.

The aim of this study was to evaluate different types of cerebral palsy and their causative risk factors by exploring prenatal, natal and post-natal history and to correlate these findings with neuroimaging findings. There is scarcity of data on neuroimaging findings in children with cerebral palsy in Pakistan. This study will also highlight potential benefits of imaging with a focus on improving etiological understanding which may help formulating management plans for preventable causes of cerebral palsy in our country.

## METHODS

The study was conducted in Pediatric OPD of Shifa Falahee Community Health center (SFCHC), Islamabad from January 2020 to July 2021. The community center serves mainly the underprivileged families and provides care at a very minimum fee, it also serves a large catchment area and receives referral from neighboring provinces. All children with disorders of movement, posture and tone diagnosed as cerebral palsy between 1-16 years were enrolled. Children with neuropathies, myopathies, metabolic and neurodegenerative diseases were excluded. A detailed history on antenatal, natal, postnatal events was taken on a predesigned form. Previous records were reviewed for NICU admission, need for phototherapy, exchange transfusions. In case where birth records were not available to check for APGAR scores, history of delayed cry was noted. Mode of delivery and place of delivery, gestational age at delivery and birth weight were noted. Complete neurological examination was performed to determine the clinical type of CP. Neuroimaging findings on CT/ MRI if done any time from birth to time of OPD visit were recorded. Informed written consent was taken from parents. Approval was taken from Institutional Review Board (Ref: IRB# 131-621-2019 dated: February 9, 2022).

Data was analyzed on SPSS version 23. Mean and standard deviation were calculated for quantitative variables like age, gestational age, height, and weight. For qualitative variables like gender, type of CP and neuroimaging findings, frequency and percentages will be calculated. P value of <0.05 will be taken as significant.

## RESULTS

A total of 89 patients were included in the study; of these 62 (69.7%) were male and 27 (30.3%) were female. Mean age was 4.4±2.8 years. Spastic variety was the most common type of cerebral palsy as shown in Table-I. Majority of babies were born at term 74 (84%) and 15 were preterm (16%). Minimum age for preterm was 30 weeks. Most of the patients were born in hospital 55 (62%) and in maternity clinics 21 (23 %), however, 13 (14%) were born at home by untrained birth attendants. Mean birth weight of our study population was 2.3 ±0.3. Lowest birth weight recorded was 1.5 kg. Out of these 89 patients, 65 (73%) were born through Spontaneous vaginal delivery (SVD), 14 (15.9%) patients were born through emergency c- section while 10 (11.4%) had elective C-sections. Mean maternal age in our cohort of patients was 26 ±6.2 years. Majority of mothers (42.7%) were primigravida.

**Table I:** Types of cerebral palsy.

Spastic	No	%
quadriplegia	48	53.9
diplegia	16	18
hemiplegia	9	10.1
monoplegic	1	1.1
Ataxic	4	4.5
Dyskinetic	2	2.2
hypotonic	9	10.1

A significant majority 38 (42.6%) had history of delayed cry suggestive of birth asphyxia. At birth 37 (41.6 %) needed neonatal intensive care unit admission. Consanguinity was seen in 56 (62.9%) and 17 (19.1%) also reported family history of cerebral palsy. Most common maternal risk factor was anemia 13 (14.6%) followed by pregnancy induced hypertension (9%) and infections (6%). No risk factors were identified in majority of mothers (57%).

Neuroimaging was done in only 37 (41.5%) patients, among these CT scans was done in 23 (25%) and MRI brain was performed in 14 (15.7%). Table-II shows neuroimaging findings in relation with type of CP and underlying etiology. Birth asphyxia 38 (42.7%) was the most common cause of CP in our cohort of patients followed by meningo-encephalitis 13 (14.7%) as shown in Table-III. Table-IV highlights risk factors identified.

**Table II:** Neuroimaging findings.

Findings	Cause	Motor type	37 (n)	%
Cerebral atrophy, encephalomalacia’ hydrocephalus	Hypoxic ischemic encephalopathy Hypotonic	Spastic quadriplegia	15	40.5
			1	2.7
Ischemic infarct	Stroke	Hypotonic	2	5.4
		Hemiplegia	1	2.7
		ataxic	1	2.7
Intracranial bleed	Intracranial bleed	Hemiplegia diplegia	2	5.4
Periventricular leukomalacia	PVL/ premature	diplegia	2	5.4
Periventricular calcification	Congenital CMV	hemiplegia	1	2.7
Gliosis, post meningitic changes	Post meningitic sequelae	Hemiplegia quadriplegia	2	5.4
Pachygyria, dysgenesis corpus callosum	Structural malformation	Diplegic Monoplegic quadriplegic	3	8.1
Cerebellar atrophy	Kernicterus	ataxic	1	2.7
Report/film not available	Idiopathic	quadriplegia	2	5.4
Reported normal	Idiopathic	Diplegia	2	5.4
		Hypotonic	1	2.7
		monoplegic	1	2.7

**Table III:** Aetiology of cerebral palsy.

Birth Asphyxia	38 (N)	42.7(%)
Idiopathic	19	21.3
Meningo encephalitis	13	14.7
Infarct/bleed	6	6.7
Prematurity	6	6.7
kernicterus	3	3.4
Structural malformation	3	3.3
Congenital CMV	1	1.1

**Table IV:** Risk Factors for cerebral palsy.

Risk factors		No (89)	%	p-value
Place of delivery	Hospital	55	61.7	0.09
	Home	13	14.6	
	Maternity clinic	21	23.5	
Mode of delivery	C Section	65	73	0.40
	SVD	24	26.9	
Birth weight	Low BW	42	47.1	0.05
	Normal BW	47	52.8	
Gestational age at delivery	Term	74	83.1	0.80
	Preterm	15	16.8	
Gender	Male	62	69.6	0.04
	Female	27	30.3	
History of delayed cry	Yes	38	42.6	<0.05
	No	51	57.3	
consanguinity	Yes	56	62.9	0.70
	No	33	37.0	
Maternal risk factors	Yes	31	34.8	<0.05
	No	58	65.1	

## DISCUSSION

Cerebral palsy is a wide spectrum neurological disorder with lifelong implications both for patient and his family.^14^ In resource limited countries like Pakistan it adds to the burden of the already constrained health care system and most of the times does not offer the holistic care cerebral palsy patients need.^15^

We enrolled 89 patients diagnosed with cerebral palsy in our community health center over a period of 13 months.^16^ The numbers are comparable with other studies which have national registries for CP. Boys were more frequently seen as compared to girls in our cohort of patients.^17^ Another study done in Karachi shows a similar gender pattern.^18^ Literature supports the fact that male gender is associated with high risk and severity of cerebral palsy, however, the cause of this association is uncertain.^19^

Maternal anemia, pregnancy induced hypertension (PIH) and infections were significantly associated with risk of CP (<0.05). Anemia has also been reported from India reflecting poor nutritional status.^20^ In contrast literature from developing countries reports maternal obesity as a risk factor for cerebral palsy in term babies.^21^ Another study from Botswana in Africa, identified maternal HIV as a significant risk factor.^22^ Antenatal and intra partum interventions are needed at a national level to decrease the maternal risk factors.^23^ Majority of mothers (42.7%) were primigravida. Consanguinity rates are high in Pakistan and 19% of the patients also had family history of cerebral palsy calling for the need of genetic evaluation and counselling.

Among the natal risk factors observed in our study, low birth weight (LBW) 15 (16%) was significantly associated with risk of cerebral palsy (<0.05). History of delayed cry was more common with SVD (57%) (p <0.05) conferring the risk of birth asphyxia. Home deliveries by untrained birth attendants (Dais) were reported in 13 (14%) of the babies. Overall, in Pakistan it is estimated that 50% of the births take place at home.^5^ This puts emphasis on the need for trained birth attendants and safe deliveries. Neonatal risk factors identified in our study are consistent with other studies in our regional countries like China.^24^

Neurological types of cerebral palsy were spastic, ataxic, dyskinetic and hypotonic. Spastic quadriplegia is the most common reported (53.9%) followed by spastic diplegia (18%). Studies from India report a similar pattern of 61% and 22% respectively.^25^ Population based study from Bangladesh shows slight predominance of hemiplegia/ monoplegia over quadriplegia 27% vs 25%.^26^ A hospital based survey done in Pakistan showed diplegia slightly more than quadriplegia 33% versus 34%.^27^

Neuroimaging was done in 37 (38.7%) of the patients only. CT scan was done more frequently in 23 (25.8%) and MRI in 14 (15.7%). This is due to wider availability and convenience of doing CT scan in children as compared to MRI. However, MRI has better detection rates in identifying brain abnormalities. Predominant abnormality noted on neuroimaging were white matter injury in the form of cortical atrophy, periventricular leukomalacia. Grey matter injury like gliosis and encephalomalacia indicative of post- meningitic sequela, vascular insults, and structural malformations in three patients. Brain abnormalities were detected in 33 (89%) of the scans, these are consistent with population based studies done on neuroimaging finding in CP in European countries. However, the number of patients who had neuroimaging done in our cohort was low reflecting the access to care. Neuroimaging helped in identifying the exact etiology and neuroanatomy of the brain insult. Neuroimaging findings were significantly correlated with etiologies (<0.05). Neuroimaging is recommended in all cases of cerebral palsy for better understanding of the neuroanatomical disease process.

Birth asphyxia leading to hypoxic ischemic insult to the developing brain is the most common cause of cerebral palsy in our patients 38 (42.7%). It presented with spastic quadriplegia (29) in majority of patients. Brain infections like meningo-encephalitis were the second leading cause 13 (14.6%). Post-meningitic sequelae presented as spastic quadriplegia in majority (nine) patients, followed by ischemic stroke and intracranial bleed 6 (6.7%). Kernicterus was reported in three patients (3.4%) two had quadriplegia while one had dyskinetic CP. Structural malformation like pachygyria, dysgenesis of corpus callosum, hydrocephalus identified on the basis of neuroimaging reports three patients presenting as quadriplegic, diplegic and monoplegic respectively. No cause could be identified in 19 (21.3%) patients. Given the high rates for consanguinity and family history of CP reported, we need to understand the genetic basis of disease. It is estimated that some 30% of CP might have genetic causation.^28^

## CONCLUSION

Prevalence of cerebral palsy is not known in Pakistan. Male gender is more affected in our cohort of patients. Maternal anemia, pregnancy induced hypertension, LBW are significant risk factor for cerebral palsy in Pakistan.

### Author’s Contribution:

**SAK:** Conceptualization, data collection, analysis, manuscript writing.

**ST:** Data collection, analysis, drafting.

**MIM:** Data analysis, drafting, final review.
